# Fox Squirrels Match Food Assessment and Cache Effort to Value and Scarcity

**DOI:** 10.1371/journal.pone.0092892

**Published:** 2014-03-26

**Authors:** Mikel M. Delgado, Molly Nicholas, Daniel J. Petrie, Lucia F. Jacobs

**Affiliations:** Department of Psychology, University of California, Berkeley, California, United States of America; University of Lethbridge, Canada

## Abstract

Scatter hoarders must allocate time to assess items for caching, and to carry and bury each cache. Such decisions should be driven by economic variables, such as the value of the individual food items, the scarcity of these items, competition for food items and risk of pilferage by conspecifics. The fox squirrel, an obligate scatter-hoarder, assesses cacheable food items using two overt movements, head flicks and paw manipulations. These behaviors allow an examination of squirrel decision processes when storing food for winter survival. We measured wild squirrels' time allocations and frequencies of assessment and investment behaviors during periods of food scarcity (summer) and abundance (fall), giving the squirrels a series of 15 items (alternating five hazelnuts and five peanuts). Assessment and investment per cache increased when resource value was higher (hazelnuts) or resources were scarcer (summer), but decreased as scarcity declined (end of sessions). This is the first study to show that assessment behaviors change in response to factors that indicate daily and seasonal resource abundance, and that these factors may interact in complex ways to affect food storing decisions. Food-storing tree squirrels may be a useful and important model species to understand the complex economic decisions made under natural conditions.

## Introduction

Food storing allows animals to take advantage of excess food in relation to current demand [1], survive periods of scarcity, and reduce foraging time during future food searches [2]. Because food-storing animals either consume items immediately or delay consumption for the future, they are a natural candidate for the study of the evolution of economic decisions such as discounting. The successful retrieval of previously stored food caches should impart a significant fitness advantage to the storer [3]. However, storing food is inherently riskier than eating it immediately as cached food may spoil, be forgotten or be pilfered by others.

Unlike general foraging decisions (e.g. [4]) which have been broadly studied, the decision to eat or cache a food item is not as well understood. This may be because many motor movements of caching are innately programmed [5], [6]. Yet even the expression of an innate program, such as a courtship display, must be allocated according to the costs and benefits of its expression in a particular context. Even if scatter hoarding movements are expressed innately, how a scatter hoarding animal decides to allocate time and energy to individual food items is significant.

Foragers may allocate responses to a food item in proportion to its value [7] or may combine prior knowledge with current sampling of food items in a Bayesian manner [8]. Food-storing animals should balance the benefits of cache investment with the risks of cache loss. They should adjust efforts to item value, their own physical condition, their current cache inventory, and the current economic climate (e.g., current food abundance and the competition for that food).

Food-storing decisions are part of a multi-step process that is sensitive to multiple aspects of food quality and the environment [9]–[12]. To adjust the cost-benefit ratio properly for a cache, the scatter hoarder should assess the value of each item efficiently, minimizing the trade-off between speed and accuracy. The cost of such assessment has been notably absent as a variable in prior models of foraging. Models of food-storing behavior indicate that an animal's ability to obtain information about the future value of food can improve food-storing decisions [13].

Assessment of food items before consumption or storage is a common and important behavior in diverse species, including primates, fish and birds [14]–[19]. Western scrub-jays (*Aphelocoma californica*), Piñon jays (*Gymnorhinus cyanocephalus*) and Stellar's jays (*Cyanocitta stelleri*) use visual cues and handle food items to determine quality before eating, caching or rejecting seeds. White-faced capuchins (*Cebus capucinus*) will touch, bite, sniff and perform extended visual inspection of figs before deciding to eat or reject [18], and stickleback fish (*Spinachia spinachia*) increase handling time and become more selective about prey items when satiated [14]. Food-storing animals should also use information gained from assessment to mitigate the energetic costs of caching by adjusting investment, e.g., time or effort spent caching, to item value.

The scatter-hoarding fox squirrel (*Sciurus niger*) conspicuously spends time handling food items before both eating and caching. Squirrels first “paw manipulate” a food item, holding it loosely in their paws and rotating it in their mouth and then “head flick,” moving the head in a rapid rotation while holding the item in the mouth [9] (Video S1). Head flicking is highly correlated with heavier, less perishable food items, and with the subsequent caching, rather than eating these items [9]. These unique behaviors likely assess the weight, probability of spoilage or other aspects of food quality [9], [20].

After assessing a food item, tree squirrels either eat or cache it, often moving to a location away from the food source and conspecifics for either process [21]–[24]. Caching by tree squirrels begins with digging. Some squirrels perform an incomplete cache (IC), where the squirrel digs but does not bury the nut, instead moving to another location to continue the cache sequence. The squirrel chooses a final cache location, and then tamps the nut with its front teeth to seat it more firmly into the ground. Finally, the squirrel uses its paws to collect items such as leaves and loose substrate to cover the cache. Squirrels vary individually in all aspects of this sequence including time spent traveling, how many ICs they perform, and time spent covering a cache [22], [25].

**Figure 1:**
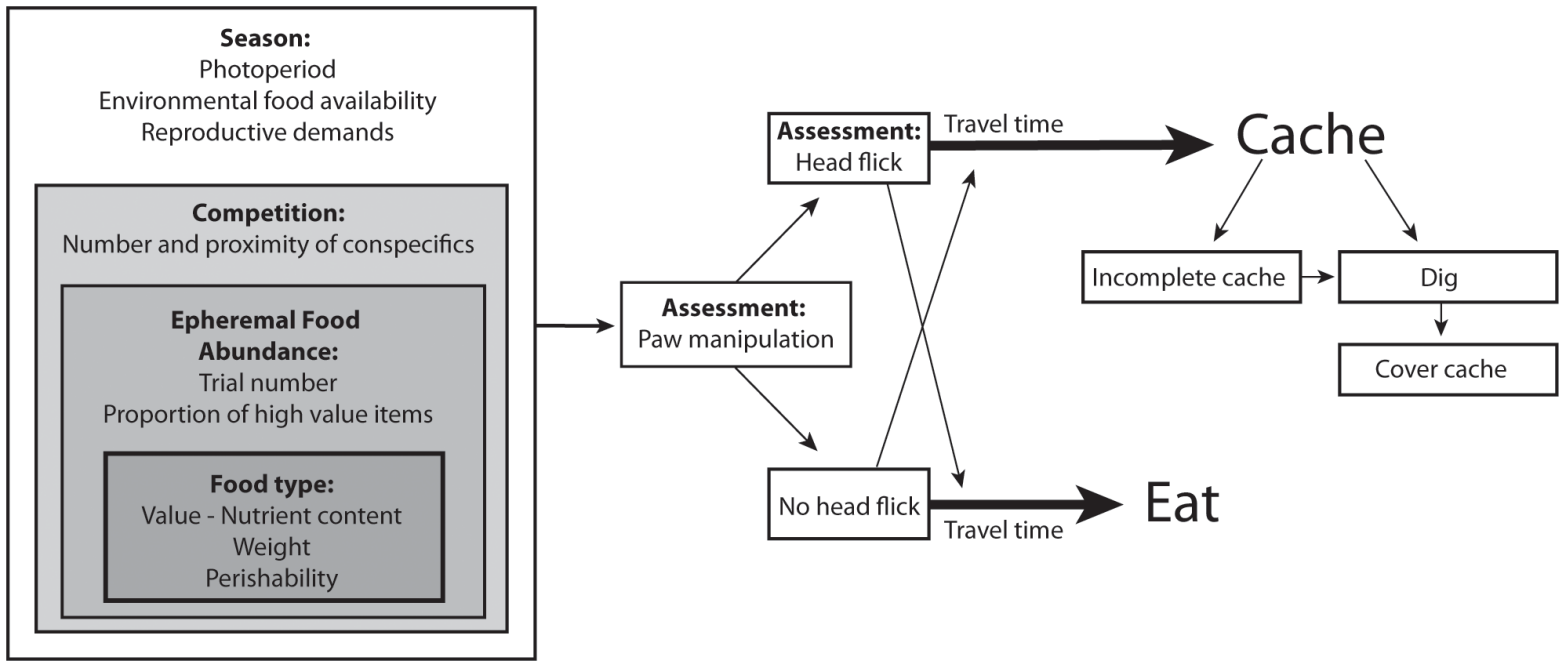
A representation of the hypothetical decision-making process in squirrels when assessing food items and investing in caches. Multiple factors (season, competition, current food availability and food type) influence assessment behaviors and cache protection strategies.

In the present study, we tested the hypothesis that free-ranging fox squirrels would adjust their assessment and investment in food items according to both intrinsic and extrinsic variables related to scarcity (Fig. 1). Fox squirrels experience seasonal fluctuations in food availability, since their largest food source is trees (e.g. oaks, hickories) [26], which produce all their seeds only in late summer and early fall and at irregular annual intervals, i.e. mast. Fox squirrels typically cache in fall and winter and retrieve the caches through the spring [20] and even summer [26]. Cached food may increase in value over time as natural food becomes less abundant and energetic costs increase in the winter [27]. We predicted that squirrels would increase their assessment and investment behaviors in the summer, when food from trees is scarce and squirrel body weights tend to be low [28]–[30].

Fox squirrels may not only be sensitive to seasonal abundance of food, but might also respond to an experimental test session as an ephemeral environmental abundance. If squirrels strictly match effort to value on an item-by-item basis, then assessment and investment should not change over trials except based on other external variables (such as nut type). However, if squirrels update their evaluation of food availability in the environment in a probabilistic or Bayesian fashion, we would expect that squirrels would decrease assessment and investment across trials, and adjust these behaviors depending on the proportion of higher-valued food items, decreasing assessment and investment when high-valued food items are more abundant within an experimental session.

Tree squirrels also modify foraging decisions in response to the value of the item [31], [32]. We expected a greater assessment of heavier, thicker-shelled, and less perishable food items (hazelnuts). Squirrels travel farther to cache larger or heavier-shelled nuts [11], perhaps to reduce pilferage by dispersing caches at a lower density [33], [34], or more likely to move them to a more open area where predation risks may deter pilferers [35]. We predicted that squirrels should invest more effort in hazelnut caches.

Finally, social pressures could affect a fox squirrel's perception of scarcity and influence its caching decisions. Eastern gray squirrels (*Sciurus carolinesis*) adjust caching behavior when facing competition, including more frequent digs and time spent on caches in the presence of other squirrels [22], [36]. We expected that fox squirrels would increase both their assessment and investment behaviors in the presence of conspecifics, relative to caches made when no other squirrels were present.

## Methods

### Study Animals

This research project was approved by the Animal Care and Use Committee of the University of California, Berkeley. The fox squirrel, an introduced species on the Berkeley campus, is well habituated to humans, allowing detailed studies of their caching behavior, memory and other decision-making processes [9], [37], [38]. Squirrels were individually marked with Nyanzol-D (American Color and Chemical Corporation, Charlotte, NC), applied from a distance by gently squirting the dye at the squirrels from a syringe. We maintained a database of marked squirrels to track individual identities.

The participants were 23 free-ranging, adult fox squirrels who were part of a larger pool of marked individuals. Ten squirrels (five female, five male) participated in the summer session, and 13 squirrels (six female, four male and three of unknown sex) participated in the fall session. Two squirrels began but did not complete the experiment (one in summer, one in fall).

### Procedure

The study site was the University of California at Berkeley campus. The site has a diversity of native and non-native food- trees, which make up at least 46% of surveyed trees in the general testing area. There at least 149 coast live oak (*Q. agrifola*), and 55 other oak trees; 55 pine trees (e.g. *Pinus pinea, Pinus ponderosa*); over 350 redwoods (*Sequoia sempervirens*), and smaller numbers of maple, hickory, walnut, hazelnut and beechnut trees (John Radke, personal communication; [39]). These trees typically peak in their mast in late September or early October [40], [41]. Furthermore, the California Acorn Survey for the time period of 2008–2013 indicated that the mast for *Q. agrifola* peaked in 2010 (Walt Koenig, personal communication; [42]). While we cannot estimate the number of acorns available to squirrels on the campus during testing, these factors indicate that the summer would have relative scarcity of food compared to the fall of this study.

As quantifying assessment required precise measurements of both counts and durations of behaviors, we videotaped and coded all sessions. We recruited squirrels with calls or gestures. The first marked squirrel to approach the experimenters was chosen to be the focal squirrel for the session. One person served as the feeder and video recorder and the second experimenter recorded the number of conspecifics in the immediate area every five minutes. All sessions were recorded using a Canon FS300 handheld camcorder.

Sessions occurred during the summer from July 22 until August 3, 2010 and during the fall from November 4 to December 2, 2010. There were two experimental conditions for handing the focal squirrel a series of 15 nuts in the shell, one at a time. We assessed how squirrels responded to receiving a sequence of 15 nuts within one session, alternating between five peanuts (a low value, highly perishable, non-native legume) and five hazelnuts (native to California; a high calorie nut with a heavy shell, and less perishable). For simplicity, we refer to both food items as nuts. In Peanut-Hazelnut-Peanut (PHP), squirrels were given a series of five peanuts, then five hazelnuts and then a second series of five peanuts. In Hazelnut-Peanut-Hazelnut (HPH), squirrels were given five hazelnuts, then five peanuts, then five hazelnuts. Peanuts weighed between 2.0 to 3.0 g, and hazelnuts weighed between 2.5 and 3.5 g. Twenty-two of 23 squirrels were tested in both experimental conditions within a season; one squirrel was tested only in one condition (“Hawaii”, male).

To determine the relative value of hazelnuts to peanuts, we calculated what percent of total nut weight was edible content for 20 peanuts and 20 hazelnuts. We found that peanuts had, on average, 73.8% (SD: 2.49%) consumable matter, while hazelnuts were 42.0% (SD: 5.17%) consumable. Taking this into consideration, we calculated the ratio of the nutritional values of each food item for the two nut types using their average weight in the study (2.5 g for peanuts, 3.0 g for hazelnuts) [43]. While per gram, hazelnuts are higher in several nutrients, per food item peanuts generally provide more calories, protein, carbohydrates, sugars, and polyunsaturated fatty acids. Peanuts and hazelnuts were similar in lipid content, and hazelnuts are higher in Vitamin E, B-6, and monounsaturated fatty acids). Hazelnuts in our study were slightly heavier, and peanuts have a soft, porous shell, which presumably makes them more susceptible to spoilage in comparison to hazelnuts.

We gave the focal squirrel the first nut of the series and then followed and videotaped them from a distance of 5 to 10 m, as they handled and either carried the nut to a cache location and completed the caching sequence, or ate the nut. After the squirrel was finished eating or caching, we gave it the next nut in the sequence. Sessions lasted between 10.58 and 47.58 minutes (*X*+SD = 25.02+7.97 minutes).

All videos of the sessions were coded using The Observer XT (Noldus, Leesburg, VA) and JWatcher 1.0 (D. Blumstein, http://www.jwatcher.ucla.edu/) by viewing videos at fifty percent speed. Files coded in JWatcher 1.0 were imported into The Observer XT for final analysis. There were five video coders, and inter-rater agreement on onset, timing and presence of behaviors ranged between 71.1 and 85.9%. We used a mutually exclusive coding scheme to record dependent variables. These included: the number of head flicks for each nut, the number of times and amount of time spent paw manipulating, time spent traveling until eating or caching, the number of incomplete caches before completing caching and the amount of time spent covering the nut. Paw manipulations could easily be discriminated from eating by observing when pieces of shell could be detected breaking away from the nut. If the squirrel ate the nut, we recorded the amount of time it took to finish consumption. We noted the outcome of every trial (eat or cache).

### Statistical Analyses

All data were analyzed using mixed models in R 2.15 (R Foundation for Statistical Computing, Vienna, Austria) and JMP 10.0 (SAS Institute, Cary, NC [44]. These models allow for repeated measures and missing data points and account for individual variability while using fewer degrees of freedom. Unless otherwise noted, Generalized Linear Mixed Models (GLMM) with a zero-inflated Poisson distribution were used to examine outcome variables that were integer-valued counts of events (number of head flicks, paw manipulation bouts and ICs), using the “glmmAMDB” package in R [45]. Logistic regression was used for the binary variable outcome (eat or cache) using the “lme4” package in R [46], [47]. JMP 10.0 was used to analyze least squares mixed models to assess continuous variables (paw manipulation time, cache time, travel time, cover time), with all variables log-transformed except for cover time, which was square-root transformed. The alpha level for all analyses was set at 0.05.

We included squirrel identity as a nominal random effect in all models, and season, nut type, condition (PHP/HPH), trial number, and whether other squirrels were present (yes/no) were included as dependent variables in all models. While we did not have a priori predictions as to how factors may interact to impact the assessment behaviors and caching decisions of squirrels, we examined all possible two way interactions of main effects as an exploratory documentation of multi-factorial decision-making in a complex environment. We removed any insignificant interaction effects in a stepwise fashion. Only significant interactions and all main effects were included in the final model to explain the effects of the dependent measures on different aspects of caching behavior.

## Results

Means (X) and standard errors (SE) are from raw data.

### Assessment Behaviors

**Figure 2:**
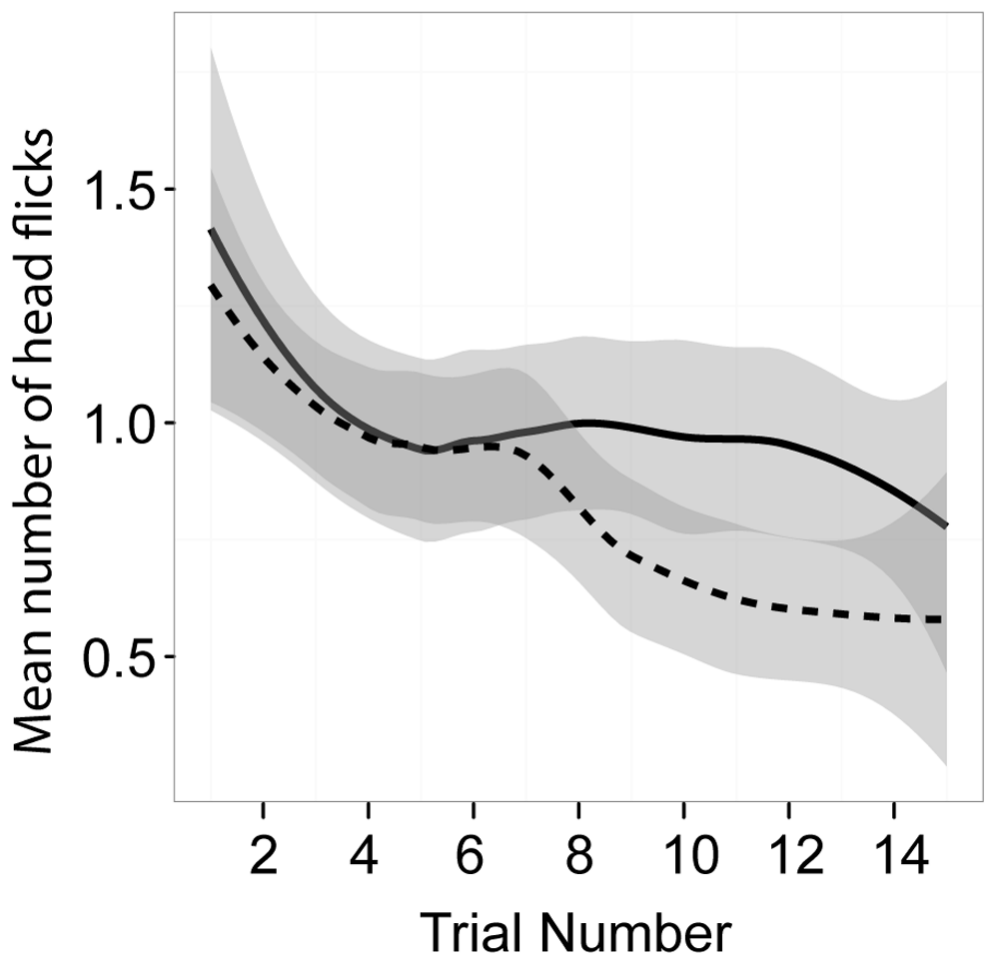
The effect of trial number on assessment. Squirrels showed a sharper decrease in head flicks across trials in the presence of other squirrels (dashed line) than no squirrels (solid line).

Squirrels performed fewer head flicks for peanuts than hazelnuts (peanuts, *X*+SE = 0.71+0.05; hazelnuts, *X*+SE = 1.05+0.06; *Z* = 4.69, *p*<.001). Squirrels showed a sharper decrease in head flicks across trials in the presence of other squirrels compared to when there were no squirrels around (*Z* = −2.35, *p* = .019) (Fig. 2).

**Figure 3:**
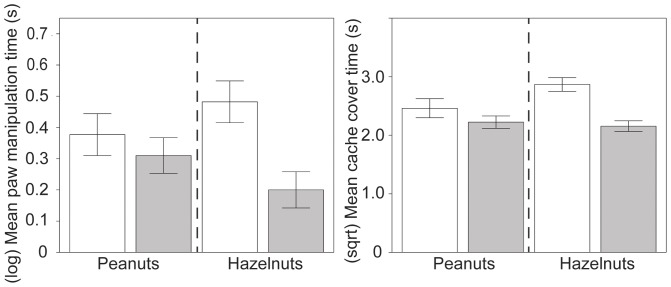
Time spent paw manipulating and covering caches by nut type and season. The increase in paw manipulations (in seconds, log transformed) and cache cover time (square root transformed) is more extreme for hazelnuts in the summer (□) compared to fall (▪). Error bars show ±1 SE.

Squirrels performed more paw manipulating bouts in the summer (*X*+SE = 1.89+0.09) than fall (*X*+SE = 1.35+0.05; *Z* = −2.91, *p* = .004) and spent more time paw manipulating in the summer (*X*+SE = 5.03+0.46 s) compared to fall (*X*+SE = 2.30+0.14 s; *F*(1, 23.81) = 4.77, *p* = .039, *r* = .41). Both paw manipulating bouts and time decreased as trials continued (*Z* = −2.98, *p*<.001). There was an interaction of nut type with season (*F*(1, 545.2) = 9.42, *p* = .002, *r* = .13), where the change in time spent paw manipulating was more extreme for hazelnuts in the summer. Time spent paw manipulating followed a U-shaped function for peanuts, and a more linear decline for hazelnuts across trials (*F*(1, 544.1) = 5.32, *p* = .021, *r* = .10) (Fig. 3).

To assess how paw manipulation was related to caching behavior, we re-analyzed the effect of the independent variables on paw manipulation only for the nuts that the squirrels cached. Squirrels spent less time paw manipulating hazelnuts (*X*+SE = 2.31+0.16 s) than peanuts (*X*+SE = 2.91+0.23 s) when caching (*F*(1, 350.7) = 13.36, *p*<.001, *r* = .19).

### Travel Time

**Figure 4:**
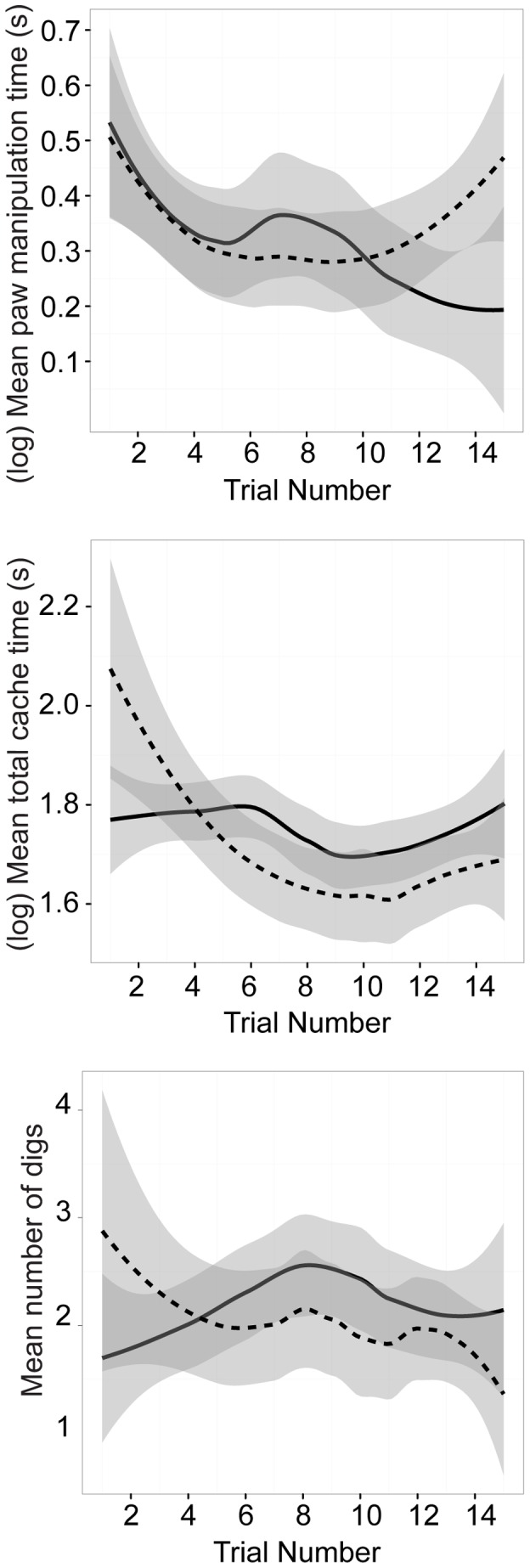
The effect of trial number and nut type on paw manipulation, cache time and ICs. The time squirrels spent paw manipulating followed a U-shaped function for peanuts (dashed line), and a more linear decline for hazelnuts (solid line) across trials. For ICs, there is an inverted U-shaped function for hazelnuts, with squirrels making more ICs toward the middle of sessions; ICs for peanuts decline across trials. The total cache time for peanuts is initially higher than hazelnuts and decreases across trials; cache time for hazelnuts is relatively consistent across trials.

**Figure 5:**
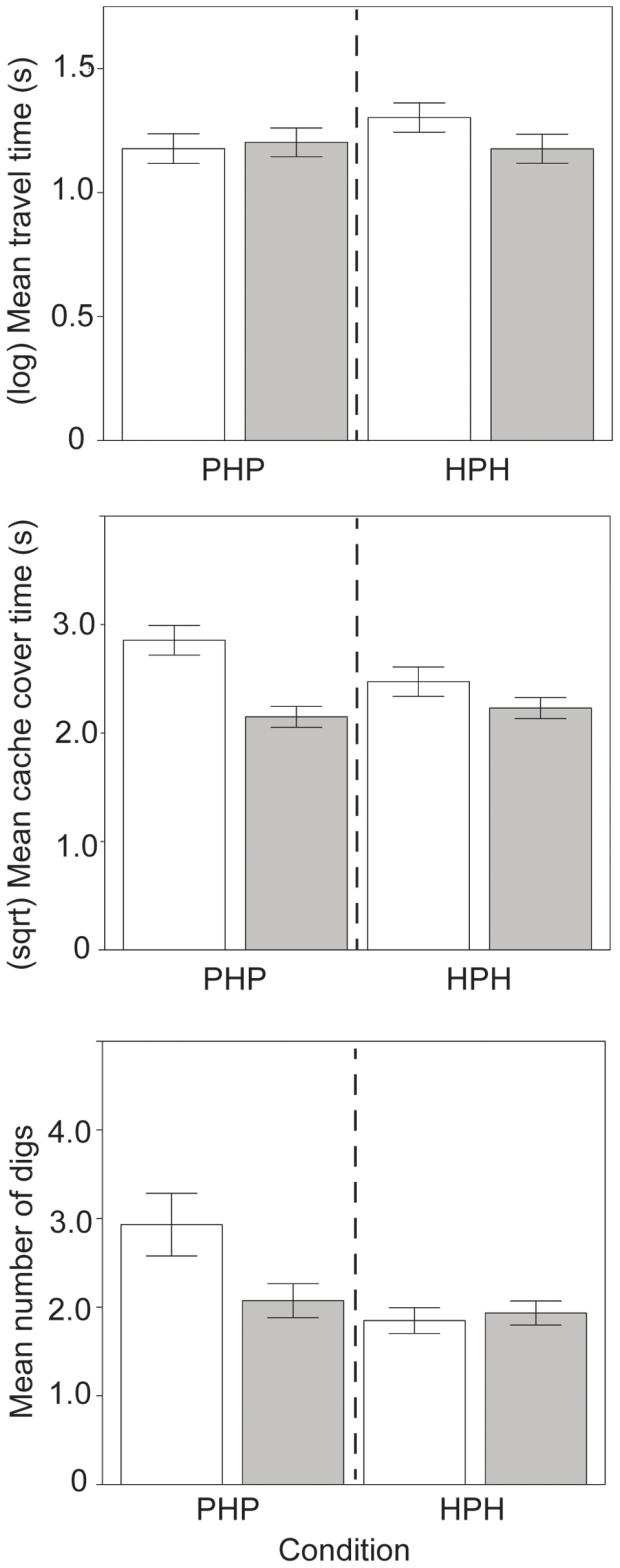
Mean time spent travelling, covering caches and number of ICs by condition and season. Squirrels spent more time travelling (log transformed) for Condition HPH (Hazelnut-Peanut-Hazelnut) in the summer (□) than fall (▪). Squirrels spend more time covering caches in Condition PHP (Peanut-Hazelnut-Peanut) in the summer, but there were no differences in cover time in fall based on Condition. Squirrels performed more ICs for condition PHP in summer. Error bars show ±1 SE.

We defined travel time as the time the squirrel moved away with the food item until they stopped to eat the nut or started digging to cache. Travel time is a reasonable proxy for travel distance (utilizing GPS, correlating straight-line distance with time traveled; Spearman's rho  = 0.62; unpublished data). Squirrels spent more time traveling to carry hazelnuts (*X*+SE = 33.6+1.86 s) than for peanuts (*X*+SE = 17.16+1.12 s; *F*(1, 559.9) = 123.51, p<.001, r = .42) but showed some tendency to increase time for peanuts as trials continued, while decreasing time traveling for hazelnuts (*F*(1, 562) = 9.67, *p* = .002, *r* = .13) (Fig. 4). Squirrels spent more time travelling in condition HPH in the summer, but there were no differences between the two conditions in the fall (*F*(1, 566.5) = 8.17, *p* = .004, *r* = .12) (Fig. 5).

### Investment in caches

**Figure 6:**
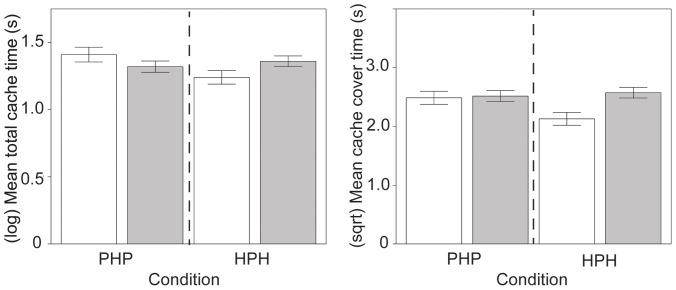
Time spent caching and covering caches under competition. Squirrels spend less time caching for condition HPH when no other squirrels were present (□). They spent less time covering caches for Condition HPH in the presence of other squirrels (▪). Error bars show ±1 SE.

Squirrels spent more time caching (from the first dig until the squirrel completed covering the cache) in summer (*X*+SE = 41.67+5.27 s) than in the fall (*X*+SE = 21.17+1.71 s, *F*(1,24.78) = 18.48, *p*<.001, *r* = .65). Squirrels decreased the time they invested in caches from trial 1 to trial 15 (*F*(1, 313.8) = 5.32, *p*<.022, *r* = .13) but this effect was not the same for both nut types (*F*(1, 313.1) = 4.23, *p* = .041, *r* = .11). The total cache time for peanuts was initially higher than hazelnuts and decreased across trials; cache time for hazelnuts was relatively consistent across trials. There was also an interaction of condition with presence of other squirrels (*F*(1, 295.2) = 7.07, *p* = .008, *r* = .15), where squirrels spent less time caching for condition HPH when no other squirrels were present (Figs. 4 and 6).

### Concealment of caches

Squirrels performed more ICs in the summer (*X*+SE = 2.47+0.20) than in the fall (*X*+SE = 1.84+0.11; *Z* = −3.25, *p* = .001). Squirrels made more ICs for hazelnuts (*X*+SE = 2.16+0.12) than peanuts (*X*+SE = 1.93+0.14; *Z* = −2.59, *p* = .010). Squirrels made fewer ICs as trials continued *(Z* = −3.04, *p* = .002) and made fewer ICs in condition HPH (*X*+SE = 1.98+0.11) compared to condition PHP (*X*+SE = 2.23+0.16; *Z* = −3.39, *p*<.001). There was a significant interaction between trial and nut type (*Z* = 3.16, *p* = .002), with an inverted U-shaped function for hazelnuts, with squirrels making more ICs toward the middle of sessions; but ICs for peanuts declined across trials. Squirrels performed more ICs for condition PHP in summer (*Z* = 2.47, *p* = .014) compared to fall, but no such difference was found for condition HPH (Fig. 5).

Squirrels spent more time covering caches in the summer (summer: *X*+SE = 8.48+0.37 s; fall *X*+SE = 5.22+0.22 s; *F*(1, 22.74) = 10.37, *p* = .004, *r* = .56) and when other squirrels were present (squirrels present: *X*+SE = 7.13+0.25 s; no squirrels: *X*+SE = 5.10+0.27 s; *F*(1, 284.3) = 7.51, *p* = .007, *r* = .16). Squirrels spent more time covering caches in condition PHP in the summer, but there were no differences in cover time in fall between conditions (*F*(1, 301.3) = 9.70, *p* = .002, *r* = .18). Squirrels also spent significantly less time covering caches for HPH in the presence of other squirrels (*F*(1, 315.6) = 7.79, *p* = .006, *r* = .16). Cache cover time was higher for hazelnuts in the summer, with a similar amount of time spent on peanuts and hazelnuts in the fall (*F*(1, 313.1) = 7.44, *p* = .0067, *r* = .15) (Figs. 3 and 6).

### Outcome

Nut type (*Z* = 5.70, *p*<0.001) and trial number (*Z* = 8.04, *p*<0.001) were both significantly related to outcome, with squirrels being more likely to eat peanuts and to cache as trials continued. Squirrels were more likely to eat in the summer (*Z* = 3.66, *p*<0.001) but the effect was dependent on nut type (*Z* = 3.10, *p* = 0.002). Squirrels cached almost all hazelnuts (99%) in the fall and most hazelnuts in the summer (76%), while they always ate more peanuts than they cached, with a more pronounced effect in the summer (78%) than the fall (59%).

Because squirrels head flick, paw manipulate and carry food items whether caching or eating, we conducted a separate analysis to examine which assessment behaviors were related to the outcome. The observation of head flicks was associated with a greater likelihood of caching (*Z* = 3.13, *p* = 0.002), with squirrels that did not head flick caching 48% of nuts, and squirrels that head flicked one or more times caching 69.8% of nuts received. Paw manipulation time was greater before eating outcomes than before caching outcomes (*Z* = −4.09, *p*<0.001). Greater travel times were associated with the caching outcome (*Z* = 7.97, *p*<0.001). The individual caching decisions of each squirrel in each condition and season are depicted in Figure S1.

## Discussion

The primary goal of our study was to determine if squirrels adjust their food assessment and cache investment behaviors in response to factors indicating scarcity. Our results suggest that squirrels are monitoring scarcity at different temporal scales (micro and macro) and that they integrate all of these factors and both scales in each decision. This is also the first examination of such a fine-grain analysis of food assessment behaviors in a wild scatter-hoarder.

Seasonal changes have predictable effects on environmental scarcity. In general, squirrels invested more per cache in the summer, when food is scarce in the environment. These caching behaviors also tended to be more variable and sensitive to the value of individual food items in the summer. For example, squirrels only increased paw manipulation time and time spent covering caches for hazelnut caches in the summer. Caching behavior becomes more stereotyped in the fall, and when large amounts of food are available, squirrels respond by caching as much and as quickly as possible, and perhaps with less deliberation and less regard for the value of individual items.

Similar patterns could be seen in squirrels' responses to smaller scale changes, i.e. within a session. Squirrels decreased paw manipulation time and bouts, cache time, and ICs as trials continued. The effect of trial number could reflect several possible factors, such as satiation, or exhaustion or behavioral discounting by decreasing investment in future rewards [48]. However, trial number influenced neither travel time nor how much time squirrels spent covering caches. As in other studies of scatter-hoarding rodents, factors in the environment may influence each step of the decision-making process in different ways [10]–[12].

The presence of other squirrels increased time spent covering caches, suggesting that squirrels created caches more carefully when social competition increased. However, squirrels also showed a rapid decline in assessment behaviors (head flicks) when conspecifics were present. This may be because assessment is costly, and a better strategy is to increase decision speed in the presence of others. Head flicking may be a signal of cacheable food and alert competitors to a potential food source, and reducing this signal in the presence of other squirrels could be a beneficial response.

In general, the influence of competition is moderated by the abundance of food [25], which perhaps explains why the effect of conspecifics in previous studies of squirrels has not been consistent, and why condition and competition interacted in their influence on cache behaviors in the present study. Previous studies have shown that eastern grey squirrels face away from others when caching food items, decrease cache density [49], increase travel time and spend more time covering caches for high-value food items when other squirrels are present [25]. In other studies, fox squirrels showed no influence of competition on number of head flicks or digs when nuts were in the shell [9] and there was no effect of conspecific presence on digging in eastern gray squirrels [25].

On the finest scale of behavioral resolution, our results show that squirrels are highly sensitive to differences in individual food items. Squirrels invested more in hazelnut caches and were more likely to head flick hazelnuts than peanuts, and food type was the only factor that influenced this behavior. Our results support previous findings that the head flick is a part of an assessment process related to nut quality, perishability, and weight [9]. Perishability is an important determinant in whether a squirrel should eat or cache a nut [50], especially given that peanuts, per food item, may have provided squirrels with more calories and were more likely to be eaten than cached. The increased tendency to cache rather than eat as trials continued may also be caused by the caloric intake from eating peanuts during the session.

Increased time paw manipulating decreased the likelihood of caching. Before eating a nut, this behavior is likely related to finding the easiest location to break into the shell. Yet squirrels also paw manipulate before caching, suggesting that squirrels may use this behavior to assess its value, search for imperfections in the shell, or to determine how best to carry it to a cache site. Future research could experimentally vary nut quality, size, texture, and portability to identify the function of paw manipulations.

In summary, our results indicate that rather than being stereotyped and invariant as food storing has been described by behavioral economists [51], squirrels adjust assessment behaviors depending on the food item and whether they will eat or cache the nut, they travel different distances depending on food value, and they alter cache protection strategies depending on food type, season and competition. Interaction effects in this study suggest that squirrels weigh environmental variables differently and may respond to multiple factors in a Bayesian manner to quantify the likelihood of scarcity, as this population of squirrels does for spatial cues [52]. Future experiments could tease apart whether these cache protection strategies differ in efficacy, and how they interact to influence behavior.

We propose that these food assessment and cache investment behaviors represent flexible economic decisions in a non-human species. For example, we found that assessment and investment behaviors decreased consistently across trials. In humans, decreased decision time when purchasing items indicates two opposing causes: impulsive and irrational decision-making versus the effect of experience and expertise [53]. In squirrels, reduced assessment could save time when a predictable source of food is available or there are competitors for food. Reduced investment across trials could be evidence for devaluation, or discounting, of food items as they continue to be available. For future studies, this could be tested by determining if a squirrel's decreased assessment and investment actually predicts lower survival of its caches, in particular those containing more valuable food items.

We temper the conclusions of our study by acknowledging that they are limited by some factors that cannot be controlled adequately in the field, including food and cache abundance and sample size. Our study only collected data in one summer and one fall of the same year, which limits the generalization of our results. Photoperiodic effects on brain and behavior must also influence seasonal changes in cache decisions [54]–[56]. Because the fate of caches is unknown, we do not know if these assessment and investment behaviors actually improved cache security and recovery. Yet the value of the present study is a demonstration that by capturing rich, detailed observations of the assessment and caching behaviors of free-ranging squirrels, the ecological and ethological factors impinging on economic decisions confirmed our a priori predictions, moreover in conditions that would be difficult, if not impossible, to replicate in a laboratory.

Our study lays the groundwork for future, more complex experimental designs with squirrels, as well as for comparisons with other food-storing and hoarding species. Laboratory pigeons (*Columba livia*), laboratory rats (*Rattus norvegicus*), and non-human primates are the most common subjects of behavioral economic studies. Based on our results and previous studies of foraging and food-storing behavior, we posit that the human-habituated urban tree squirrel may be a better species than any of these as a model system to understand the interaction between extrinsic factors (such as resource availability and food value) and intrinsic factors (such as satiation and discounting) on the ecological function and evolution of economic decisions.

## Supporting Information

Figure S1 **Assessment behaviors in fox squirrels.** Squirrels paw manipulate a food item by rotating it in their mouth and paws. They secure the nut in their mouth and head flick, rapidly rotating their head back and forth.
(TIF)

Figure S2 **All cache decisions for each squirrel in the study by season and condition.** The top line for each squirrel represents peanuts (□) and hazelnuts (▪) that were eaten, the bottom line represents nuts that were cached. The top section for each squirrel is Condition PHP, the bottom section is Condition HPH.
(TIF)

Video S1 (MP4)
