# Supplementary figures and images for: Fox Squirrels Match Food Assessment and Cache Effort to Value and Scarcity
Source: PLoS One. 2014 Mar 26;9(3):e92892. doi: 10.1371/journal.pone.0092892 (PMC3966826; doi:10.1371/journal.pone.0092892)

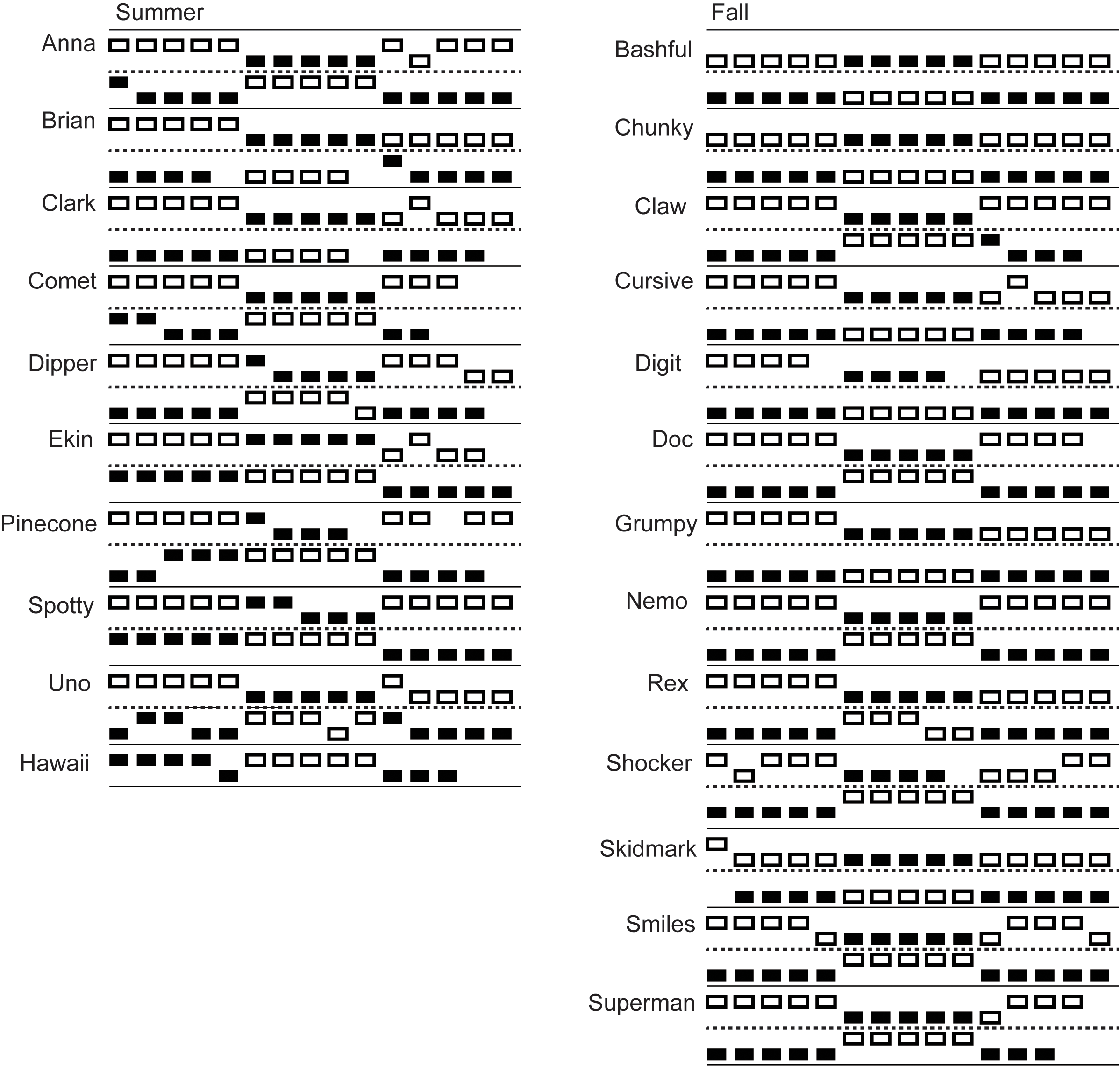

Supplement: Figure S1 — Assessment behaviors in fox squirrels. Squirrels paw manipulate a food item by rotating it in their mouth and paws. They secure the nut in their mouth and head flick, rapidly rotating their head back and forth. (TIF) [file pone.0092892.s001.tif]

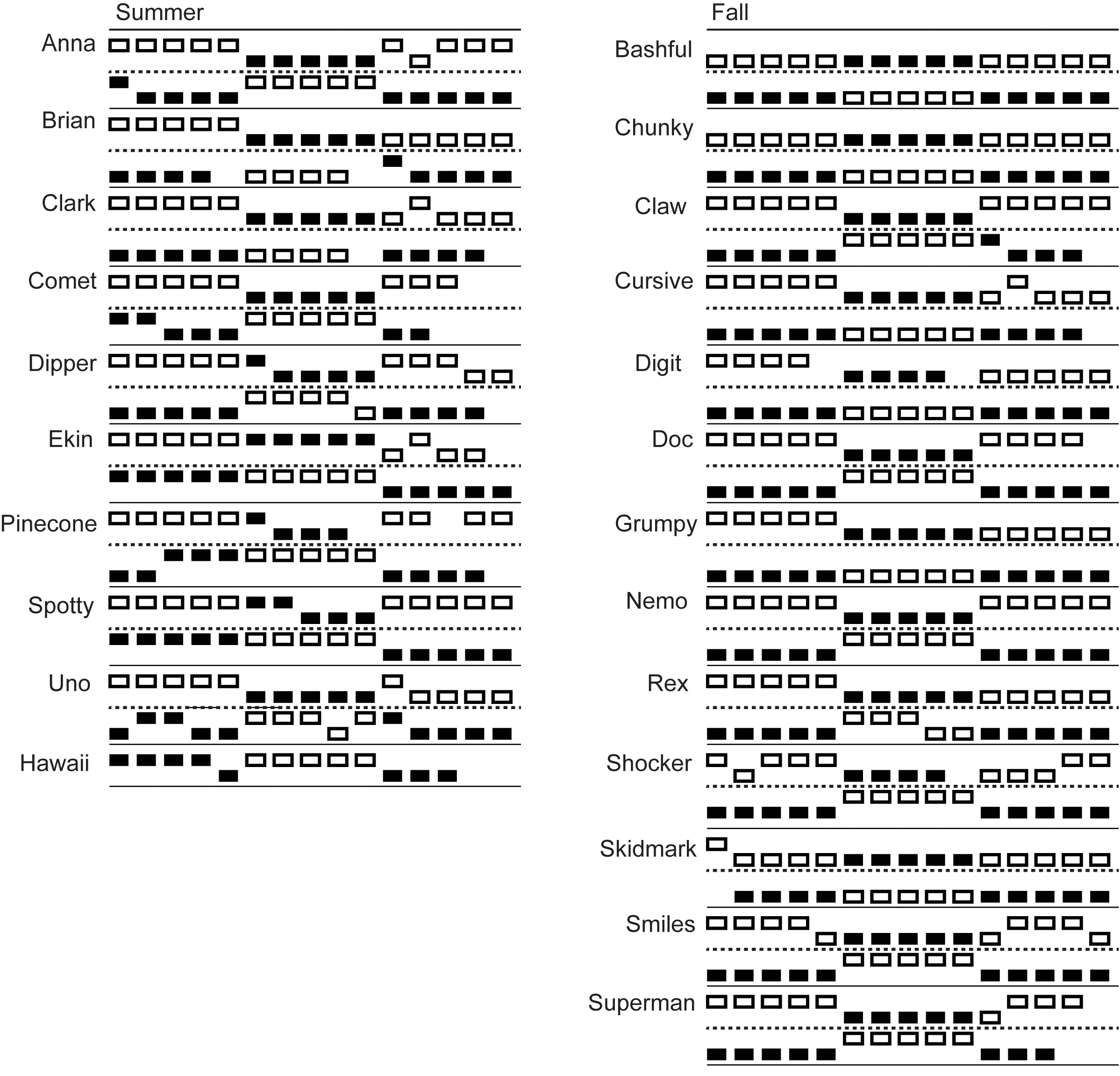

Supplement: Figure S2 — All cache decisions for each squirrel in the study by season and condition. The top line for each squirrel represents peanuts (□) and hazelnuts (▪) that were eaten, the bottom line represents nuts that were cached. The top section for each squirrel is Condition PHP, the bottom section is Condition HPH. (TIF) [file pone.0092892.s002.tif]
